# Evaluation of Anti-*Helicobacter pylori* IgG Antibodies for the Detection of *Helicobacter pylori* Infection in Different Populations

**DOI:** 10.3390/diagnostics12051214

**Published:** 2022-05-12

**Authors:** Jin-Han Yu, Ying Zhao, Xiao-Feng Wang, Ying-Chun Xu

**Affiliations:** 1 Department of Laboratory Medicine, State Key Laboratory of Complex Severe and Rare Diseases, Peking Union Medical College Hospital, Chinese Academy of Medical Sciences and Peking Union Medical College, Beijing 100730, China; pumc_yujinhan@student.pumc.edu.cn (J.-H.Y.); 13681200202@163.com (X.-F.W.); xycpumch@139.com (Y.-C.X.); 2 Graduate School, Chinese Academy of Medical Sciences and Peking Union Medical College, Beijing 100730, China

**Keywords:** *Helicobacter pylori*, immunoassay, IgG antibodies, physical examination population, allergic diseases

## Abstract

Serological testing (immunoassay) for *Helicobacter pylori* (*H. pylori*) is widely available and inexpensive, and does not require medication modifications before testing. It can also determine the type of infection, which helps with clinical diagnosis and treatment, and guides the use of medication. However, the performance of immunoblotting for the detection of *H. pylori* infections in different populations has still not been fully evaluated. We performed a retrospective analysis of patients in the Health Examination Center and Outpatient Department, from November 2017 to September 2020, at Peking Union Medical College Hospital. All the subjects were tested with the ^13^C-urea breath test (^13^C-UBT) and for IgG antibodies. A total of 1678 participants, including 1377 individuals who had undergone physical examinations, were recruited. The results of the immunoassay were significantly different from those of the ^13^C-UBT for all the subjects and outpatients (*p* < 0.001). For the physical examinations of individuals, the agreement between the immunoassay and the ^13^C-UBT was 0.64 (95%CI: 0.59–0.68; *p* < 0.001), and the *H. pylori* immunoassay demonstrated a sensitivity and specificity of 74.24% and 90.45%, respectively, with a positive predictive value of 71.01% and negative predictive value of 91.76%. In addition, in patients with gastric mucosal atrophy or early gastric cancer, antibody typing tests can also detect infected patients with missed UBT. The prevalence of *H. pylori* in Beijing was 26.8%, and the serological positivity rate for *H. pylori* in the population of Beijing was about 31.7% (25.1% in the physical examination population). The rate of *H. pylori* antibody positivity among patients with allergic diseases was 73.5%, which is significantly higher than that of the non-allergic disease population (29.3%, *p* < 0.001). In conclusion, *H. pylori* antibody typing testing can be applied as a specific test in the healthy physical examination population, and the test can be performed with the remaining serum during the physical examination.

## 1. Introduction

*Helicobacter pylori* (*H. pylori*), a Gram-negative, microaerobic bacterium with demanding growth conditions, was first successfully isolated from gastric mucosal biopsies of patients with chronic active gastritis, and it is the only microbial species known to be able to survive in the human stomach [1]. *H. pylori* infection is an important pathogenic factor in digestive system diseases (e.g., peptic ulcers and chronic active gastritis) and is strongly associated with stomach cancer [1,2]. Nearly half of the world’s population is currently infected with *H. pylori*, and the International Agency for Research on Cancer has also listed *H. pylori* (infection) on its list of class I carcinogens [3].

In the genes encoding *H. pylori*’s virulence factors, which contribute to different degrees of pathogenicity of strains, very high heterogeneity and genetic variation have been found [4]. Cytotoxin-associated gene A (*cagA*), one of the most studied genes with a virulence-associated function, encodes the CagA effector protein, which is delivered into gastric epithelial cells through the secretion of bacterial type IV, which is an oncoprotein that induces malignant neoplasms in mammals [5,6]. Another well-known virulence factor of *H. pylori* is vacuolating cytotoxin A (VacA). This is a toxin secreted by the type Va secretion system (T5aSS) and is known to induce the formation of cytoplasmic vacuoles [5]. Moreover, urease is produced by *H. pylori* and catalyzes urea hydrolysis, producing the end products of carbon dioxide (CO_2_) and ammonia (NH_3_), facilitating survival in hostile pH conditions, and improving settlement and growth in the human gastric epithelium [7]. Urease A (UreA) and urease B (UreB) are the two structural subunits of urease heterodimers [7]. To resist *H. pylori* infection, the body produces corresponding anti-virulence-factor antibodies, which are also helpful for auxiliary diagnosis. Urease is the main antigenic component for antibody production in *H. pylori* infection. Interestingly, UreB can induce multiple autoimmune diseases by stimulating B-1 cells to generate self-reactive antibodies, e.g., IgG3, IgM-type rheumatoid factors, and anti-single stranded DNA (ssDNA) [7,8,9].

The prevalence of *H. pylori* varies greatly by region of the country, from a relatively low prevalence of 20%, to 50% in high-income areas, and to as high as 80% or more in low-income areas [10]. Gastric cancer is the third most common cause of cancer deaths around the world, and *H. pylori* infection is the single strongest risk factor [10,11,12]. Each year, approximately 340,000 people in China suffer gastric cancer due to *H. pylori* infection [10]. The main methods used to diagnose *H. pylori* infection include (i) invasive operations to obtain mucosal tissue for histopathology and/or various molecular and nucleic acid amplification tests, and (ii) non-invasive operations such as urea breath tests, stool antigen tests, and serological tests [13,14].

The ^13^C-UBT, as a gold-standard method, has been in use for over 30 years and is the most widely used and accurate non-invasive test for the diagnosis of *H. pylori* infection worldwide, with advantages such as high accuracy in *H. pylori* detection, easy operation, and not being influenced by the focal distribution of *H. pylori* in the stomach [13,14]. Unfortunately, the ^13^C-UBT is severely limited in patients with a history of recent use of acid-suppressant or antibacterial agents, acute upper gastrointestinal bleeding, accelerated gastric emptying due to gastrectomy or gastric acid deficiency, bile reflux, and severe atrophy/enterocolitis of the gastric mucosa [5,14,15].

Since individuals infected with *H. pylori* develop a local and systemic immune response, specific *H. pylori* antibodies can be detected by rapid serological assays. Serological assays, as non-invasive methods, are simple, rapid, and inexpensive, and enable immediate patient testing for *H. pylori* antibodies in general practice surgeries [9]. The serological test can be used for some specific conditions (peptic ulcer bleeding, gastric MALT lymphoma, and severe gastric atrophy) [15]. A number of fast *H. pylori* antibody tests are commercially available; however, the clinical value of the immunoassays in different populaces has not been adequately assessed.

This study aimed to investigate the epidemiology of *H. pylori* infection in the Beijing area and evaluate the clinical significance of the serum antibody typing of *H. pylori* in Chinese outpatients and the physical examination population.

## 2. Materials and Methods

In this study, we performed a retrospective analysis of all the patients in the Health Examination Center and Outpatient Department who had undergone both *H. pylori* antibody typing testing (serum) and ^13^C-UBT, from November 2017 to September 2020, at Peking Union Medical College Hospital. This study was approved by the ethics committee of Peking Union Medical College Hospital (ethical approval number: S-K2069).

### 2.1. ^13^C-UBT

The ^13^C-UBT was performed using breath test analyzer HCBT-01 and a ^13^C-UBT Kit (Shenzhen Zhonghe Headway Bio-Sci & Tech Co., Ltd., Guangdong, China). Briefly, breath samples were obtained, after 12 h of fasting, before (baseline) and 30 min after the intake of the Headway ^13^C-urea capsule in the early morning. The ^13^CO_2_ and ^12^CO_2_ concentrations were measured separately using the breath test analyzer, and then, we calculated the DOB (‰) of the change in ^13^C isotopic abundance versus the natural abundance of ^12^C in the sample and at baseline. A sample was considered positive if the 30 min value was above a 4‰ cut-off level. Eating, drinking, and smoking were not permitted until the ^13^C-UBT was finished. All the subjects stopped using proton-pump inhibitors (PPIs), H2-receptor antagonists, and other acid suppressants for two weeks before the test, and stopped using antibacterial drugs, bismuth-based drugs, and certain herbal medicines with antibacterial effects for four weeks before the test.

### 2.2. H. pylori Antibody Typing Testing (Serum)

A qualitative Western blot kit assay, with an *H. pylori* antibody Immunoblotting Kit (Shenzhen braute Biological Products Co., Ltd., Guangdong, China), was utilized to assay IgG antibodies. The test protocol was as follows: *H. pylori* antigens were electrophoresed on a sodium dodecyl sulfate (SDS)–polyacrylamide gel, separated by sub-atomic loads, and then transferred to nitrocellulose membranes. The anti-*H. pylori* antibodies present in the serum responded to the antigens on the nitrocellulose membrane and were visualized with the expansion of enzyme-labeled antigens and shading reagents. A positive zone appeared as shading on the membrane. A negative result occurred when the quality-control zone showed up on the shading-rendering zone; a type 1 *H. pylori* immune response was observed when the CagA or VacA zone, or both, appeared, and a type 2 *H. pylori* immune response was observed when the UreA or UreB zone, or both, appeared and the CagA and VacA zone did not appear.

### 2.3. Statistical Analysis

All the experimental data were analyzed with the SPSS Statistics 26.0 software. The clinical performance of the *H. pylori* immunoassay was evaluated by determining the sensitivity, specificity, positive predictive value (PPV), and negative predictive value (NPV) compared to the ^13^C-UBT. McNemar’s test was utilized to analyze the sensitivities and specificities for the assessment of the two diagnostic tests. The agreement between the two tests was determined utilizing Cohen’s kappa (κ) with 95% certainty stretches (CI). The degree of statistical significance was set at *p* < 0.05.

## 3. Results

### 3.1. General Characteristics of the Study Population

A total of 1678 participants, including 1377 physical examination individuals, underwent the *H. pylori* immunoassay and ^13^C-UBT test, of which 1276 (76.0%) were male and 402 (24.0%) were female. The median age was 37 (range: 3–91) years. The *H. pylori* ^13^C-UBT positivity rate was 26.8% in all the subjects, with a significant difference between sexes (*p* = 0.026); the rates were 25.5% in males and 31.1% in females. The type 1 *H. pylori*-positivity rates were 25.2% and 25.1% in males and females, respectively, whereas more females than males were positive for type 2 *H. pylori* (11.9% versus 5.1%, respectively). Among the 1230 participants who had a negative ^13^C-UBT, 10.4% (*n* = 128) tested positive for type 1 *H. pylori*, and 4.7% (*n* = 58) tested positive for type 2 *H. pylori*. Furthermore, a history of previous *H. pylori* infections was recorded in 29.5% of the medical records. The main population demographics and antibody patterns are provided in Table 1.

Among the patients diagnosed with *H. pylori* infection according to clinical physicians (no clinical diagnosis information for the physical examination population), the seroprevalence was 80.6% and the ^13^C-UBT positivity rate was 59.7%. Interestingly, the proportion of allergic diseases (6.0%) in the population is similar to that of patients with digestive system diseases (6.5%). However, among the patients diagnosed with allergic diseases, the positivity rate for the *H. pylori* ^13^C-UBT test was 48.0%; 57.0% of allergic diseases patients were infected with type 1 *H. pylori*, and 15.0%, with type 2 *H. pylori*. On the contrary, in the group of patients with digestive system diseases, only 26.6% of the patients were ^13^C-UBT positive; type 1 *H. pylori* accounted for 29.4%, and type 2 *H. pylori* accounted for 16.5% (Table 1).

### 3.2. Comparison of H. pylori ^13^C-UBT Positivity Rates among Different Age Groups

**Figure 1:**
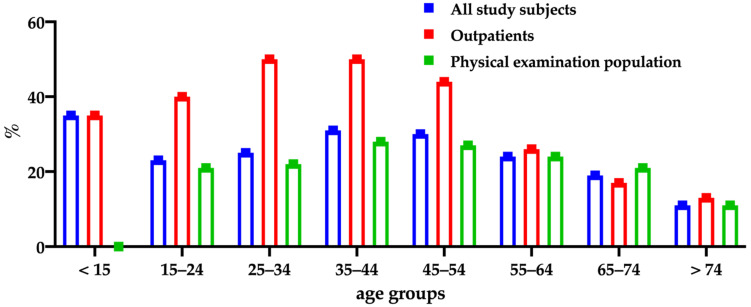
*H. pylori* ^13^C-UBT positivity rates among different age groups.

The *H. pylori* positivity rates in the different age groups of <15, 15–24, 25–34, 35–44, 45–54, 55–64, 65–74, and >74 years were 35.3%, 22.8%, 25.2%, 31.3%, 30.4%, 24.3%, 18.8%, and 11.1%, respectively. There were significant differences (*p* = 0.033) among the age groups. The positivity rates were the highest in the 35-to-44-years group in both the immunoassays and ^13^C-UBT (Figure 1).

### 3.3. Performance of H. pylori Antibody Typing Testing

In testing for *H. pylori,* the ^13^C-UBT serves as the gold standard [9,13,14]. The results for the immunoassay were significantly different from those of the ^13^C-UBT for all the subjects and outpatients (*p* < 0.001). It is worth noting that, for physical examination individuals, the agreement between the immunoassay and the ^13^C-UBT was 0.64 (95%CI: 0.59–0.68; *p* < 0.001), and the *H. pylori* immunoassay demonstrated a sensitivity and specificity of 74.24% and 90.45%, respectively, with a positive predictive value (PPV) of 71.01% and a negative predictive value (NPV) of 91.76% (Table 2).

### 3.4. Antibody Patterns in Different Populations

**Figure 2:**
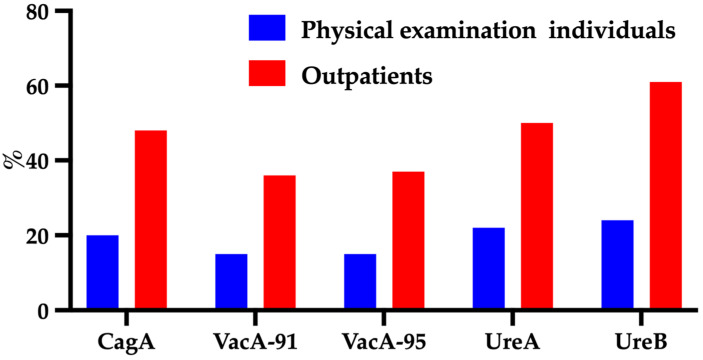
Distribution of antibody typing in outpatients and physical examination individuals. Vac-91 and Vac-95 represent antibodies with molecular weights of 91 and 95 KD, respectively.

A comparison of the positivity rates for the five antibodies in all the subjects revealed that the outpatients’ seropositivity rate was higher than that of the physical examination subjects (Figure 2).

**Figure 3:**
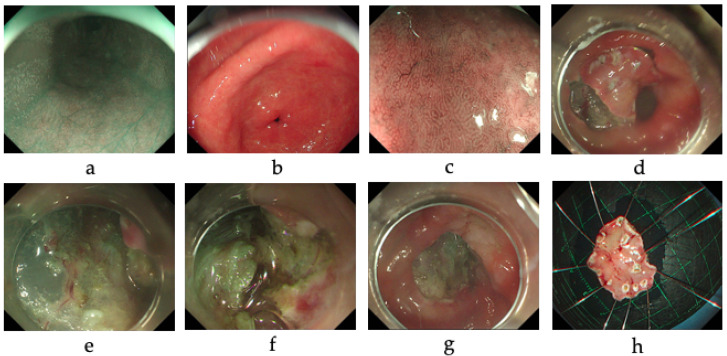
Patient 12, with high-grade intraepithelial neoplasia of the gastric mucosa, tested positive on the immunoassay, stool antigen tests, and rapid urase test, and negative in the UBT. (**a**–**c**) The esophageal mucosa was smooth and pink, with no erosions, ulcers, or varices seen, and the dentate line was clear. The cardia and the mucosa of the fundus were not appreciable abnormalities. The mucosa of the gastric body was red-white and reddish, and no obvious ulcers and neoplasia were seen; the mucosa of the gastric horn was scattered thin white, mildly enteric manifestation, and no obvious ulcers and erosions were seen. (**d**–**g**) A mucosal lesion (Ila + IIc) with a diameter of about 0.5–1.0 cm was seen on the less curved side of the anterior pyloric region of the gastric sinus, with localized post-biopsy changes and clear borders, as shown by indigo carmine staining. The lesion was circumferentially marked with a DualKnife and circumferentially incised, and the lesion was peeled along the submucosa, with no adhesions in the submucosa and no significant bleeding. (**h**) The size of the specimen was about 2.5 × 2 cm as measured in vitro, and the size of the lesion was 0.5 × 0.5 cm.

Among the patients with digestive system diseases, 29.4% were positive for CagA antibodies, and 22.0%, for VacA antibodies. In addition, 33.0% of the patients were positive for UreA antibodies, while 45.9% were positive for UreB antibodies. The results of the UBT and antibody typing tests in 14 patients with clinically diagnosed gastrointestinal tumors are shown in Table 3. All the patients were treated with endoscopic submucosal dissection (ESD). Only Patient 12, with high-grade intraepithelial neoplasia (HGIN) of the gastric mucosa, tested positive for all five antibodies, based on stool antigen tests and a rapid urase test. Unexpectedly, this patient was negative for ^13^C-UBT. The results of the electronic gastroscopy report for Patient 12 are shown in Figure 3. Another three patients with early gastric cancer and with chronic atrophic gastritis were positive for urease antibodies and had negative UBT tests. In addition, both Patient 3 and Patient 6 were antibody-negative within a year and a half, during which time the UBT test was consistently negative. Two other patients with early gastric cancer tested positive for ^13^C-UBT only, and no infection was detected by immunoassay.

The rate of *H. pylori* antibody positivity among patients with allergic diseases is 73.5%, which is significantly higher than that of the non-allergic disease population (29.3%, *p* < 0.001). Of concern to us was that 69% of the patients with allergic diseases were positive for UreB antibodies, 57% for CagA, 43% for VacA, and 57% for UreA antibodies. The positivity rates for CagA, VacA, UreA, and UreB in patients with allergic diseases who tested positive in the UBT were 76.1%, 67.4%, 87.0%, and 78.3%, respectively. Of these, only four patients were negative for all the antibodies (clinical diagnosis of allergic rhinitis and allergic bronchopulmonary aspergillosis, allergic rhinitis and urticaria, antiphospholipid antibody syndrome, and urticaria, respectively).

## 4. Discussion

Gastric cancer remains the third leading cause of cancer mortality, with more than half of all the gastric cancer cases in the world occurring in East Asia, primarily in China [10,11,12]. A crucial strategy for preventing gastric cancer in China, as well as other high-risk regions worldwide, is population-wide screening for and eradication of *H. pylori* [16]. The preferred test for *H. pylori* is ^13^C-UBT, but the equipment required to perform this test is expensive and requires regular maintenance, and the test is also prone to false negatives for people with upper gastrointestinal bleeding or those taking acid suppressants [13,14,15]. Immunoblotting for *H. pylori* antibodies is inexpensive, laboratory-operated, and drug-independent, and the fifth Chinese National Consensus Report on the management of *Helicobacter pylori* infections has also recommended that immunoassays can be performed for patients with bleeding ulcers and diseases associated with low bacterial density (extensive mucosal atrophy and mucosa-associated lymphoid tissue lymphoma) [5,15]. However, the accuracy of immunoblotting for the detection of *H. pylori* infections in different populations had still not been fully evaluated.

^13^C-UBT is considered to be the most robust non-invasive gold-standard method for the detection of *H. pylori* [9,13,14]. In our study, whether for all the study subjects or only outpatients, the results of *H. pylori* antibody typing testing (serum) were significantly different from the ^13^C-UBT results. However, given that the two methods for the physical examination population had good consistency (95%CI: 0.59–0.68; *p* < 0.001), the sensitivity and specificity of the *H. pylori* antibody typing testing were 74.24% and 90.45%, respectively, with a PPV of 71.01% and NPV of 91.76%. The results of this study further suggest that serological testing is more appropriate for the health-screening population. In primary care hospitals or low-income remote areas, where ^13^C-UBT testing is not available, serum samples left over from the health-screening process could be used to screen for *H. pylori* infections.

Among the patients clinically diagnosed with *H. pylori* infections, the seroprevalence (80.6%) was significantly higher than the ^13^C-UBT positivity rate (59.7%). The percentage of people who tested positive for *H. pylori* only by serum and were negative in the ^13^C-UBT was 11.1%. It is widely known that, after the successful eradication of *H. pylori*, it may require longer than 1 year for the *H. pylori* antibodies to disappear [17]; a positive antibody test may only indicate a prior infection. However, it is also possible that atrophic gastritis due to severe infection may not be effectively detected by UBT, as in our abovementioned patient with early gastric cancer, who was positive in serology and fecal antigen testing but negative in the UBT alone. In this case, the antibody typing testing is a better auxiliary to the diagnosis of *H. pylori* infection.

A study of the physical examination population of Guangxi, China, showed significant differences among various age groups [18]. We also found that the *H. pylori* positivity rates in the different age groups showed significant differences (*p* = 0.033). However, the positivity rate in the ^13^C-UBT and antibodies among people older than 45 years tended to decline with age (Figure 1).

Studies have proposed that infection rates vary by geographic region [3]. A cross-sectional population study of 1797 individuals showed a *H. pylori* seroprevalence of 48% in Germany [19]. Enko et al. [9] used a commercially available immunoassay for 108 patients, and the seroprevalence was 45.4%. In the study of Guangxi, the seroprevalence of *H. pylori* infections was 58.3% [18]. However, in our study of immunoassays at Peking Union Medical College Hospital, the serological positivity rate was 31.7%. The antibody positivity rate was 25.1% if only the physical examination population was considered, and 63.1% if only outpatients were taken into account. In conclusion, the prevalence of *H. pylori* in Beijing is low, regardless of whether the whole population or only physical examination patients are considered. In addition, the prevalence of *H. pylori* infection by gender was also significantly different (*p* = 0.026); this is consistent with previous research that found that the prevalence of *H. pylori* infection was higher in females than in males [20].

CagA is one of the most well-studied virulence factors of *H. pylori*, a toxin encoded by *cagA* and carried by cag-PAI. It is known that the *H. pylori* strain that carries the PAI is more virulent than the strains that do not [21]. Some studies have suggested that higher serum anti-CagA IgG titers are significantly linked to gastric mucosal inflammation, and this marker can be considered a risk factor for the progression of gastric cancer [15,22]. The second important toxin in the repertoire of *H. pylori* virulence factors is VacA. All the type 1 *H. pylori* cases in our study carried the *CagA* gene. The limited data for digestive tumor patients in our study did not reveal a significant association between the vacA genotype or cagA and gastric cancer, and the results of the Chinese Macau study also concluded that the two aforementioned virulence factors were not associated with the development of gastric cancer [23].

The involvement of *H. pylori* infection in many extra-gastroduodenal manifestations remains a fascinating field of investigation. *H. pylori* infection might be related to such extra-gastric diseases as rheumatoid arthritis, Sjögren’s syndrome, systemic lupus erythematosus, iron-deficiency anemia, and mucosa-associated lymphoid tissue lymphoma [2,8]. The latest research also shows that *H. pylori* infection is connected with an increased risk of growth disorders in children and the progression of atherosclerotic disease [2,24]. Serum IgG should be the first choice for studying the long-term relationship between *H. pylori* infection and growth in children [2]. In our study, patients with allergic diseases had significantly higher rates of *H. pylori* seropositivity than patients with non-allergic diseases. A previous study also found that, in the Western world and some developing countries, the rates of *H. pylori* infection in children and adults were declining, in marked contrast to the increase in asthma and allergic diseases in children [25]. From 1983 to 2018, *H. pylori*’s prevalence declined by 0.9% annually in China [26], while the prevalence of asthma in China increased rapidly [27], which indicates that a similar allergic disease and *H. pylori* infection phenomenon also exists in China.

The major limitation of this study was that the study population was taken from a single center. However, Beijing is the political and economic center of China, with a diverse urban population, and in addition, Peking Union Medical College Hospital is a national center for the diagnosis and treatment of difficult and serious diseases appointed by the National Health Commission, so the results of the study can be considered generalizable to areas (the prevalence is about 30%) with a relatively lower prevalence.

## 5. Conclusions

The results of the *H. pylori* antibody typing test (serum) were in excellent agreement with those of the UBT test in the health-screening population, and they had a high specificity. In addition, in patients with gastric mucosal atrophy or early gastric cancer, antibody typing tests can also detect infected patients with missed UBT. The prevalence of *H. pylori* in Beijing is 26.8%, and the serological positivity rate for *H. pylori* in the population of Beijing, China, is about 31.7% (25.1% in the physical examination population), which is low compared to that in other regions. The prevalence of *H. pylori* antibodies in patients with allergic diseases is significantly higher than that in patients with non-allergic diseases, and in recent years, China has gradually shown a trend of decreasing prevalence of *H. pylori* and increasing prevalence of allergic diseases, as in Western countries.

## Figures and Tables

**Table 1:** Basic characteristics and antibody patterns of the study population.

Variable	Frequency (%)	^13^C-UBT+	Type 1 *H. pylori*	Type 2 *H. pylori*
Sex (*N* = 1678)				
Male	1276 (76.0)	325	321	65
Female	402 (24.0)	125	101	48
Median age (years)	37 (range: 3–91)	41 (range: 3–91)	41 (range: 4–91)	42 (range: 19–77)
Only *H. pylori* seropositive	186 (11.1)	/	128	58
Classification of diseases ^†^				
Allergic diseases	100 (6.0)	48	57	15
Digestive diseases *	109 (6.5)	29	32	18
*H. pylori* infection	67 (4.0)	40	43	11
Stomach tumors	17 (1.0)	2	1	3

^†^ Physical examination individuals generally had no clinical diagnosis, and duplicate patients were not excluded. * Digestive diseases include patients with *H. pylori* infections and stomach tumors.

**Table 2:** *H. pylori* antibody typing testing versus ^13^C-UBT in different populations.

	All Subjects ^†^ (*n* = 1678)		Outpatients ^‡^ (*n* = 301)		Physical Examination ^§^ (*n* = 1377)	
	*H. pylori* antibody+	*H. pylori* antibody−	*H. pylori* antibody+	*H. pylori* antibody−	*H. pylori* antibody+	*H. pylori* antibody−
^13^C-UBT +	346 (20.6%)	102 (6.1%)	106 (35.2%)	14 (4.7%)	245 (17.8%)	85 (6.2%)
^13^C-UBT −	186 (11.1%)	1044 (62.2%)	84 (27.9%)	97 (32.2%)	100 (7.3%)	947 (68.8%)

^†^ All the study subjects, including the physical examination population; ^‡^ Only outpatients; ^§^ Only the physical examination population.

**Table 3:** The results of UBT and *H. pylori* antibody typing testing (serum) in 14 patients with clinically diagnosed gastrointestinal tumors.

	Sex	Age (Years)	Diagnosis	Sampling Time	CagA	VacA (91 KD)	VacA (95 KD)	UreB	UreA	^13^C-UBT
Patient 1	Male	51	HGIN (IIb); chronic atrophic gastritis	17 January 2019	Neg	Neg	Neg	Neg	Neg	Neg
		52		5 May 2020	Neg	Neg	Neg	Neg	Neg	Neg
Patient 2	Female	57	HGIN; chronic superficial gastritis	25 May 2020	Neg	Neg	Neg	Neg	Neg	Neg
Patient 3	Female	65	HGI (IIa + IIc); chronic non-atrophic gastritis	21 March 2019	Neg	Neg	Neg	Pos	Pos	Neg
		66		18 May 2020	Neg	Neg	Neg	Neg	Neg	Neg
Patient 4	Male	75	HGIN	25 July 2019	Neg	Neg	Neg	Neg	Neg	Neg
Patient 5	Male	64	After ESD; chronic atrophic gastritis	22 March 2019	Neg	Neg	Neg	Neg	Neg	Neg
Patient 6	Female	71	HGI (IIa + IIc); chronic atrophic gastritis	9 May 2019	Neg	Neg	Neg	Pos	Pos	Neg
		72		24 August 2020	Neg	Neg	Neg	Neg	Neg	Neg
Patient 7	Male	46	HGI (IIa + IIc); chronic atrophic gastritis	25 April 2019	Neg	Neg	Neg	Neg	Neg	Neg
Patient 8	Male	47	HGI (IIa + IIc); chronic atrophic gastritis	23 April 2020	Neg	Neg	Neg	Neg	Neg	Neg
Patient 9	Female	66	Gastric sinus mucosal lesions; *H. pylori* infection	20 April 2020	Neg	Neg	Neg	Neg	Neg	Pos
Patient 10	Male	68	HGIN; *H. pylori infection*; chronic atrophic gastritis	17 January 2019	Neg	Neg	Neg	Pos	Neg	Neg
Patient 11	Male	74	HGIN; chronic superficial gastritis	26 December 2019	Neg	Neg	Neg	Neg	Neg	Neg
Patient 12	Male	53	HGIN (IIa + IIc); *H. pylori* infection; chronic atrophic gastritis	10 January 2019	Pos	Pos	Pos	Pos	Pos	Neg
Patient 13	Male	33	HGI (IIa + IIc); *H. pylori* infection	3 February 2020	Neg	Neg	Neg	Neg	Neg	Pos
Patient 14	Male	65	HGI (IIb + IIc); Chronic atrophic gastritis; Reflux esophagitis	23 September 2019	Neg	Neg	Neg	Neg	Neg	Neg

## Data Availability

Not applicable.

## Abbreviations

*H. pylori*	*Helicobacter pylori*
IgG	Immunoglobulin G
^13^C-UBT	^13^C-urea breath test
cagA	Cytotoxin-associated gene A
VacA	Vacuolating cytotoxin A
UreA	Urease A
UreB	Urease B
NPV	Negative predictive value
PPV	Positive predictive value
CI	Confidence intervals
PPI	Proton-pump inhibitors
ESD	Endoscopic submucosal dissection
